# Sickness absence in relation to first childbirth in nulliparous women, employed in the education and care branches in the public or private sectors: A Swedish longitudinal cohort study

**DOI:** 10.1371/journal.pone.0274603

**Published:** 2022-09-15

**Authors:** Krisztina D. László, Pia Svedberg, Petra Lindfors, Ulrik Lidwall, Kristina Alexanderson

**Affiliations:** 1 Division of Insurance Medicine, Department of Clinical Neuroscience, Karolinska Institutet, Stockholm, Sweden; 2 Department of Global Public Health, Karolinska Institutet, Stockholm, Sweden; 3 Department of Psychology, Stockholm University, Stockholm, Sweden; 4 Department for Analysis and Forecast, Swedish Social Insurance Agency, Stockholm, Sweden; National Cheng Kung University College of Medicine, TAIWAN

## Abstract

**Background:**

Pregnancy and childbirth entail increased risks of sickness absence (SA). Many women work in education and care, two branches characterised by high SA levels; it is not known if the link between childbirth and SA in these branches differs between private and public sectors. We examined SA and disability pension (DP) in relation to childbirth among women working in the education and care branches, and if these patterns differed between public and private sectors.

**Methods:**

We performed a Swedish register-based cohort study. Study participants were nulliparous women living in Sweden in December 2004 and employed in education or care (n = 120,013). We compared SA/DP in the three years before and after 2005 among women who had no childbirth during follow-up (B0), had one childbirth in 2005 and no more (B1), and had one childbirth in 2005 and at least one more during follow-up (B1+). Analyses were performed for all and by public or private sector.

**Results:**

Of all studied women, 70% worked in the public sector. Women in B1 and B1+ had, except for the year before childbirth, comparable or lower mean combined SA/DP days than women in the B0 group; women in the B1+ group had, except for the year before childbirth, the lowest mean level of SA/DP. We observed no substantial differences in these patterns between public and private sectors.

**Conclusions:**

Patterns of SA/DP among nulliparous women who did or did not give birth did not differ substantially between public and private sectors among women in the educational and care branches.

## Introduction

Several studies suggest that pregnancy and childbirth are associated with a temporary increase in sickness absence (SA) [1–3]. In several Swedish twin and population-based studies, women who gave birth were found to have higher combined mean SA and disability pension (DP) days during the year proceeding childbirth than their nulliparous counterparts [1, 3–6]. However, women who gave birth had lower levels of SA/DP in other periods before and after birth than women who did not give birth, with SA levels being lowest among women who had more than one childbirth [1, 3–6].

SA levels are known to be determined not only by morbidity but also by factors such as the physical and psychosocial work environment, level of work demands, private-life circumstances, labor-market conditions, attitudes towards SA and the social insurance system [7–11]. In Sweden, the labor market is characterized by a high gender segregation, both regarding workplaces and occupations [12, 13] which may play a role for SA [14].

Overall, the education and care branches have among the highest levels of SA [15–18]. Both these branches and their main occupations are numerically dominated by women. For example, 91% of assistant nurses and of those working in elderly care, 89% of nurses, 89% of childcare staff, 75% of daycare teachers, and 72% of personal assistants are women [19, 20]. Suggested explanations for the high SA in the education and care branches involve adverse working conditions with high emotional demands, heavy physical load (e.g., heavy lifting or strenuous work positions), or low skill levels [6, 16, 21–25]. Further, according to absence culture theories, several female-dominated occupations may be more flexible with respect to possibilities to combine work and private life and more permissive with respect to reasons and levels of work absence [26, 27]. In contrast, certain occupations in education and care, e.g., physicians or teachers, may be prone to sickness presenteeism, due to perceived difficulties to identify short-term substitutes when on SA or due to concerns about adding extra burden to their colleagues; however, sickness presenteeism may result in longer SA spells later [28].

In Sweden, women working in the educational and care branches are predominantly employed in the public sector, i.e., by municipalities that are responsible for providing primary and secondary education and child and elderly care, or by counties, that organize healthcare. Both education and care branches can be organized by public or private organizations, even if mainly publicly financed. Levels of SA/DP in Sweden are higher in the public than in the private sector [29]; the explanations for this inter-sectorial difference are not fully understood but may relate to 1) better work organization, psychosocial, and physical working conditions in the private than in the public sector [29, 30]; 2) contextual factors such as organizational size and geographical location; 3) differences in the composition of the sectors’ workforce with respect to gender, age, occupation, etc.; and 4) the health selection that results from these three aspects, as well as other ones [31, 32]. Work tasks within education and care are often highly regulated, thus any differences after having considered the workforce composition between the public and private sectors may be indicative of differences in the work organization and certain working conditions [33]. Most studies on the topic, include all employed, that is, does not differentiate with regard to childbirth. To our knowledge, no previous study has investigated patterns of SA/DP before and after childbirth among women in relation to giving birth or not, in the education and care branches and potential differences between public or private sectors.

The aims of this study were to analyze 1) levels of SA and DP during the years before and after first childbirth, and among women with no childbirth in the education and care branches and 2) whether the levels differed between private and public sectors.

## Materials and methods

A population-based longitudinal cohort study was conducted, based on nationwide register microdata. A cohort used in previous studies [4, 6, 34] was used to identify the cohort for this study, including all nulliparous women aged 18–39 years, residents in Sweden in 2002–2004, and employed in the education (preschool through high school) and care (i.e., healthcare, child care, elderly care, etc.) branches. We identified women by means of the Longitudinal Integration Database for Health Insurance and Labor Market Studies (LISA) [35] kept by Statistics Sweden and by the Medical Birth Register (MBR) and the National Patient Register (NPR), both kept by the Swedish Board of Health and Welfare. We also linked the cohort to the Cause of Death Register to identify deaths during the study period. Linkage between registers was possible by means of the unique personal identification number assigned to each Swedish resident [36].

The MBR, established in 1973 and covering 97–99% of births in Sweden, was used to obtain information on childbirths, including data on date of birth and parity. To increase coverage on childbirths, we also used the NPR to obtain information on births not found in the MBR. We searched for hospitalizations with a main or a secondary diagnosis related to child delivery, as previously described [6, 34]. Information on parity was not available for those identified from NPR. Using available information on childbirths (from 1964 in NPR and 1973 in MBR, in both registers through 2009), we created the following three childbirth groups:

- Women who had no childbirth registered neither before, nor during the three years and 43 weeks after the index date (T_0_) (“B0”).
- Women having their first childbirth in 2005, and no childbirths during the three years and 43 weeks after T_0_ (“B1”).
- Women having their first childbirth in 2005, and at least one more childbirth during the three years and 43 weeks after T_0_ (“B1+”).

For women who had their first childbirth in 2005, the date of delivery was used as T_0_. For women who did not give birth, T_0_ was set to July 2, 2005. The study period was the three years prior T_0_ and the three years after T_0_ (Y_-3_-Y_+3_), altogether six years. The additional 43 weeks were considered to account for SA or DP during a possible new pregnancy and to exclude those from the B0 group who were pregnant towards the end of the follow-up.

Information on occupation in December 2004, coded according to the Swedish Standard Classification of Occupations 1996 (SSYK 1996), the Swedish version of ISCO-88, was obtained from LISA. If information on occupation was missing in 2004, we used information from 2005. We identified women working in the education and care branches using the following SSYK codes: 1227, 1317, 232, 233, 234, 235, 33, and 3462 for education and 1228, 1318, 222, 223, 249, 322, 323, 324, 3461, and 513 for care. Since actual care activities or teaching at pre-university level is performed generally in organizations that are run by the municipalities, the county councils, and other non-state owned private or public organizations, we excluded state-employed women. Our cohort consisted of 120,013 women.

Ethical approval for the project was granted by the Regional Ethical Review Board of Stockholm (2007/762-31, 2009/23-32, 2009/1917-32, 2011/806-32, and 2016/1533-32).

### Public sickness absence and disability pension benefits in Sweden

Data on SA and DP days was obtained from the Social Insurance Agency MIDAS register [37]. In Sweden, all residents aged 16 years or older with income from work or unemployment benefits and who are unable to work due to disease or injury, are entitled to SA benefits from the public sickness insurance system [6, 38]. The employer provides sick pay for the first two weeks of a SA spell, thereafter, the Social Insurance Agency. Thus, information on SA spells ≤14 days was not available for us. All residents aged 19–64 years can be granted DP if having permanent or long-term work incapacity due to disease or injury. Both SA and DP can be granted for full- or part-time (25%, 50%, 75%, 100%) of ordinary work hours. SA benefits cover about 80% and DP about 65% of lost income, both up to a certain limit.

Pregnant women having strenuous or risky working conditions that cannot be adjusted, can apply for “pregnancy benefits” during the 10–60 days before expected delivery date. All pregnant women, regardless of their working conditions, may use up to 60 days paid “parental-leave benefits” (of the possible 480 days) during the eighth and ninth months of gestation. Both these types of benefits were at the same levels as SA benefits, additional parental-leave days with lower benefits were also possible. Parental-leave days could be used over several years, and by both parents. Working parents may also receive benefits when absent to care for a sick child. Information on pregnancy- or parental-leave benefits was not included in this study, nor any other social welfare benefits, only SA and DP.

### Sociodemographics

All sociodemographic variables were measured at 31 December 2004. The following variables were considered: age (coded as18-24, 25–29, 30–34, and 35–39 years); country of birth (Sweden, other Nordic country, other EU25, rest of the world); type of place of residence (based on the H-classification scheme [39]), large city (Stockholm, Gothenburg, Malmö), medium-sized city (>90,000 inhabitants), and small city/village (<90,000 inhabitants); educational level (elementary school (≤9 years), high school (10–12 years), and university/college (>12 years)); and family situation (dichotomized as married/cohabitant and single).

### Statistical analyses

We used two different measures of SA/DP. First, we calculated mean number of SA and DP net days per year, during the three years prior to T_0_ until three years after T_0_ for each of the three childbirth groups; both crude and standardized means were calculated. We did direct standardization using the B1 group as the standardization reference and the sociodemographic measures as standardization variables. We used the *PROC MIXED* procedure in SAS with the *LSMEAN* and *95% CI* options to estimate the standardized means and 95% confidence intervals, in separate models with SA, DP, and SA+DP as outcomes. In the standardization, all sociodemographic variables were accounted for; age, country of birth, type of place of residence, and educational level (including them as binary). Part-time SA/DP days were combined, e.g., two days of half-time SA or DP were counted as one net day. The other measure was the prevalence of having at least one SA spell or DP/year. Women who emigrated or died after T_0_ were censored after the year of emigration/death. We performed analyses for all the women in the three groups (B0, B1, and B1+) and also stratified by public versus private sector.

All analyses were conducted using SAS 9.4.

## Results

Of the 120,013 women included in the cohort, 4686 (3.9%) had their first child in 2005 and no more births in the next three years (B1), 9263 (7.7%) women had their first birth and an additional delivery during follow-up (B1+), whereas the majority (106,064; 88.4%) had no child deliveries at all prior to or during the study period (B0) (Table 1). Compared to their counterparts who did not give birth (B0), women who gave birth during the study period were more likely to be at least 25 years old, to have higher education, to be married or cohabiting, and to work in the public sector.

**Table 1:** Demographic characteristics of the study population, by childbirth status.

Variables	N (%)		
	B0 (n = 106,064)	B1 (n = 4686)	B1+ (n = 9263)
**Age (in years)**			
**18–24**	55,929 (52.73)	1134 (24.20)	1968 (21.25)
**25–29**	24,413 (23.02)	1755 (37.45)	4495 (48.53)
**30–34**	14,112 (13.31)	1273 (27.17)	2430 (26.23)
**35–39**	11,610 (10.95)	524 (11.18)	370 (3.99)
**Country of birth**			
**Sweden**	94,223 (88.84)	4108 (87.67)	8558 (92.39)
**Other Nordic country**	1181 (1.11)	63 (1.34)	92 (0.99)
**Other European country**	1209 (1.14)	49 (1.05)	77 (0.83)
**Rest of the world**	9451 (8.91)	466 (9.94)	536 (5.79)
**Type of living area**			
**Large cities**	40,870 (38.53)	1698 (36.24)	3264 (35.24)
**Medium-sized cities**	40,099 (37.81)	1709 (36.47)	3576 (38.61)
**Small cities**	25,095 (23.66)	1279 (27.29)	2423 (26.16)
**Educational attainment**			
**Elementary (≤9 years)**	11,546 (10.89)	309 (6.59)	369 (3.98)
**High school (10–12 years)**	48,498 (45.73)	1991 (42.49)	2877 (31.06)
**University/college (≥13 years)**	46,020 (43.39)	2386 (50.92)	6017 (64.96)
**Family situation**			
**Married or cohabitant**	5697 (5.37)	1022 (21.81)	2498 (26.97)
**Single**	100,367 (94.63)	3664 (78.19)	6765 (73.03)
**Employer**			
**Public**	74,178 (69.94)	3588 (76.57)	7297 (78.78)
**Private**	31,886 (30.06)	1098 (23.43)	1966 (21.22)

B0 = No childbirth up to 2005, nor during the follow-up, B1 = First childbirth in 2005 and no more childbirths during follow-up, B1+ = First childbirth in 2005 and at least one more during follow-up.

**Fig 1:**
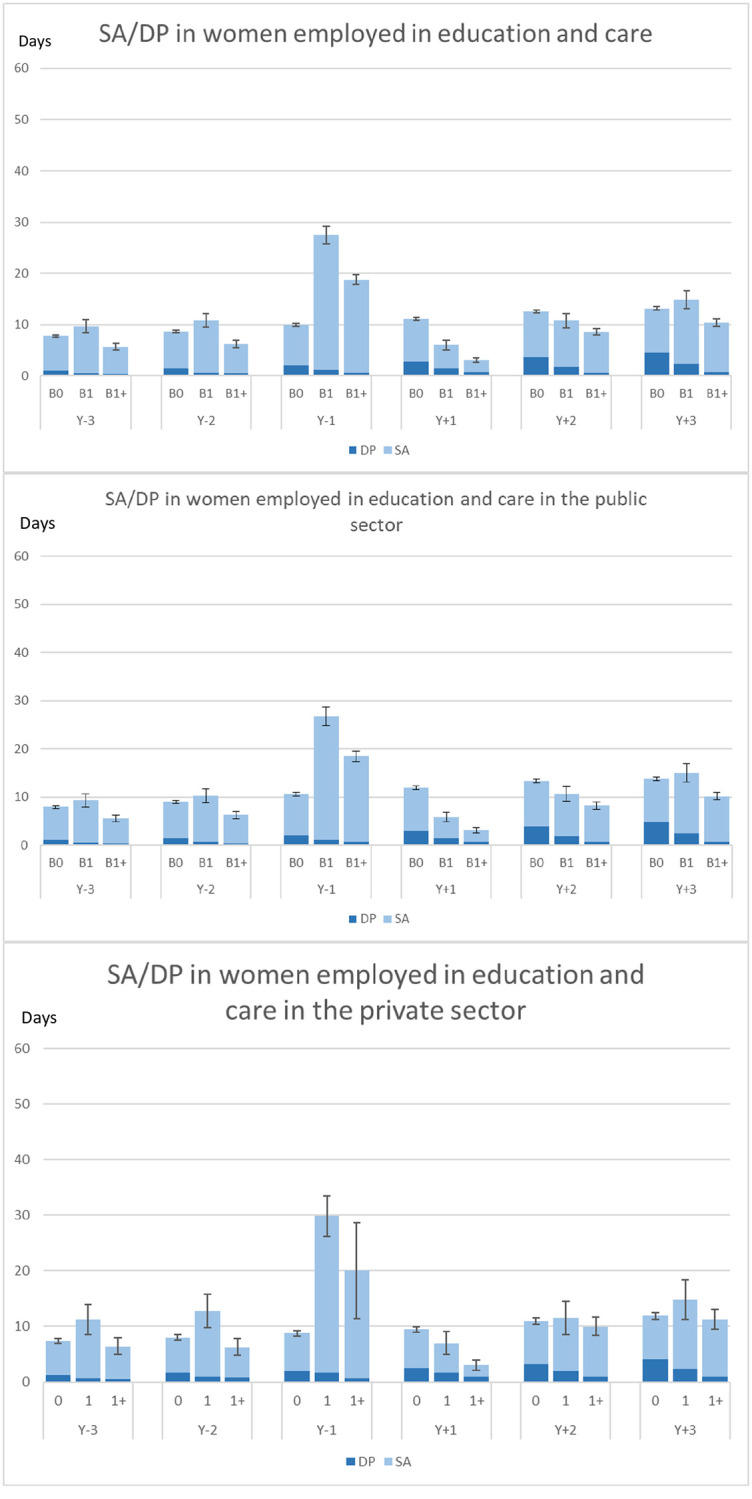
Crude mean sickness absence (SA) and disability pension (DP) net days for each of the six studied years, by childbirth status, in the total cohort, and among women working in the public sector and among women working in the private sector, respectively, among the women employed in the education and care branches. (The black bars indicate the 95% confidence intervals for the sum of SA/DP days). B0 = No childbirth, B1 = First childbirth in 2005 and no more childbirths during follow-up; B1 = First childbirth in 2005 and at least one more during follow-up. Y_-n_ = n years before the index date; Y_+n_ = n years after the index date (index date = birth date for B1 and B1+ and 2 July 2005 for B0).

**Fig 2:**
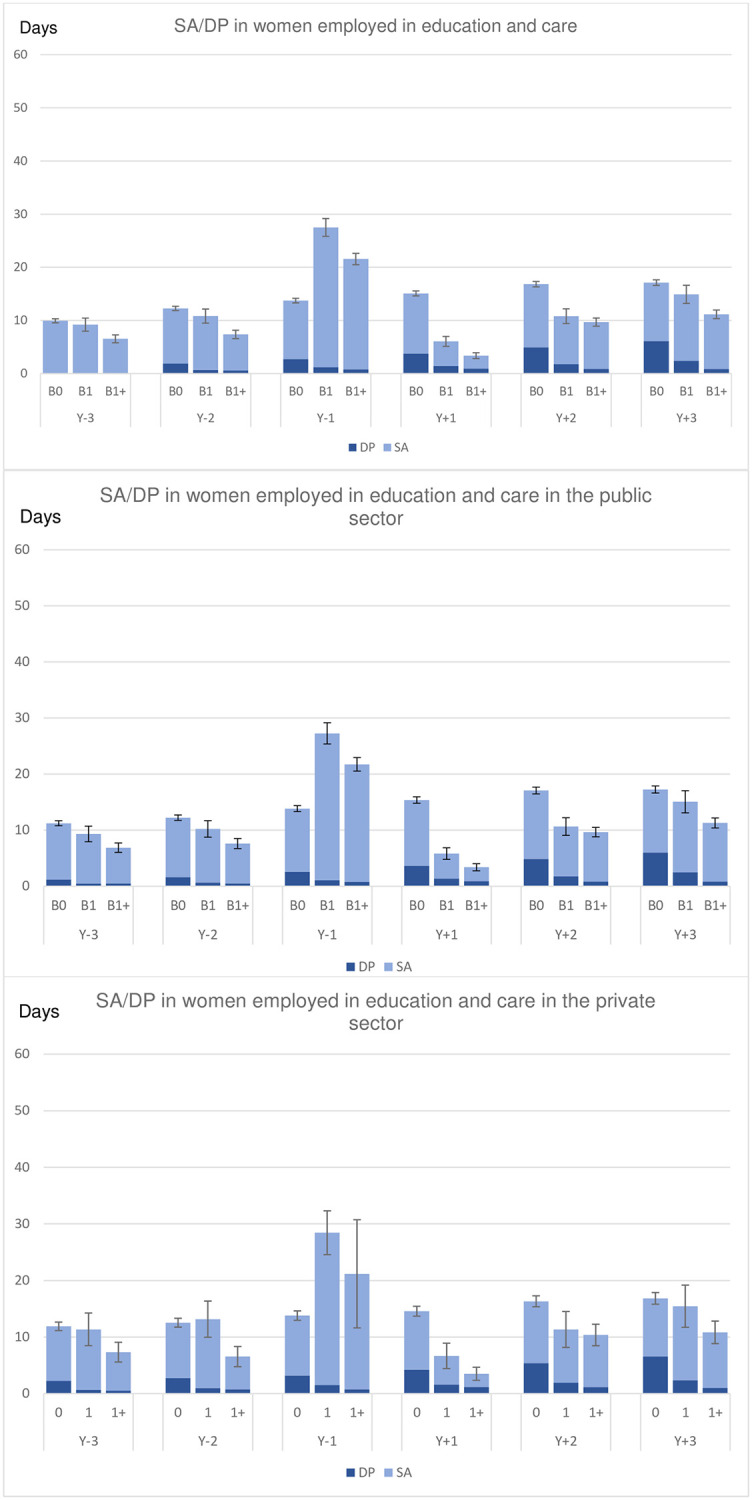
Standardized mean sickness absence (SA) and disability pension (DP) net days for each of the six studied years, by childbirth status, in the total cohort, and among women working in the public sector and among women working in the private sector, respectively, among women employed in the education and care branches. (The black bars indicate the 95% confidence intervals for the sum of SA/DP days). B0 = No childbirth, B1 = First childbirth in 2005 and no more childbirths during follow-up; B1 = First childbirth in 2005 and at least one more during follow-up. Y_-n_ = n years before the index date; Y_+n_ = n years after the index date (index date = birth date for B1 and B1+ and 2 July 2005 for B0).

In Y_-1_, that is, when pregnant, women in the B1 and B1+ groups had higher combined mean SA and DP days than those in the B0 group (Figs 1 and 2). In Y_+1_, both B1 and B1+ groups had much lower SA days, probably due to being on parental leave. Except for in Y_-1_, women in the B1 group had comparable or lower combined crude and standardized SA and DP days than women in the B0 group while the women in the B1+ group had the lowest mean level of SA/DP days. The women in B0 had a slight increase in number of SA/DP days over the six years. We observed no substantial differences in these patterns of SA/DP by childbirth group over time between women working in the public or in the private sectors.

When using the SA/DP measure of having had at least some SA/DP during a year, it was clear that the absolute majority in all three childbirth groups, had no SA spells >14 days nor any DP during the six studied years (Table 2). Even in Y_-1_, the year the women in B1 and B1+ were pregnant, the proportions with no SA/DP were 62% and 67%, respectively. The proportions of any SA/DP were, for most years, lowest among the women in the B0 group; in the years Y_-3_ through Y_+1_ women in B1 group generally had the highest proportions. However, in the years Y_+2_ and Y_+3_ women in B1+ generally had the highest proportion of having at least one SA/DP spell. The pattern of the results was similar when stratifying women by public and private sectors.

**Table 2:** Proportion of women with sickness absence and/or disability pension during the six study years, by childbirth status, in the overall cohort and by sector.

Year	Childbirth group	All (%)	By sector	
			Public sector (%)	Private sector (%)
**Y**_**-3**_	**B0**	8.16	8.48	7.40
	**B1**	12.29	12.24	12.48
	**B1+**	8.96	8.87	9.31
**Y**_**-2**_	**B0**	7.91	8.20	7.24
	**B1**	11.78	11.68	12.11
	**B1+**	8.80	8.80	8.80
**Y**_**-1**_	**B0**	8.87	9.40	7.65
	**B1**	38.45	37.82	40.53
	**B1+**	33.13	32.81	34.33
**Y**_**+1**_	**B0**	10.03	10.63	8.63
	**B1**	10.09	9.81	11.02
	**B1+**	6.49	6.55	6.26
**Y**_**+2**_	**B0**	10.73	11.38	9.22
	**B1**	10.83	10.91	10.59
	**B1+**	16.39	16.38	16.43
**Y**_**+3**_	**B0**	10.62	11.24	9.20
	**B1**	13.33	13.68	12.18
	**B1+**	20.24	20.41	19.58

B0 = No childbirth, B1 = First childbirth in 2005 and no more childbirths during follow-up; B1 = First childbirth in 2005 and at least one more during follow-up. Y_-n_ = n years before the index date; Y_+n_ = n years after the index date (index date = birth date for B1 and B1+ and 2 July 2005 for B0).

## Discussion

In this large cohort of women employed in the education and care branches, we found that women who gave birth to one child during the study period (B1) had, except for the year before childbirth, comparable or lower mean number of SA/DP days than women who remained nulliparous (B0). Moreover, the women who gave birth to more than one child during our study period (B1+) had, except for the year before childbirth, the lowest mean number of SA/DP days. We observed no substantial differences in these patterns among women working in the public or private sectors.

To our knowledge this is the first study to investigate SA/DP levels specifically among nulliparous women employed in the education and care branches around the time of the first childbirth and to analyze whether these patterns differ between those employed in the public and private sectors. Our findings regarding higher SA/DP during the year before birth among parous women compared to those who remained nulliparous corroborate the results of previous studies–including several from our group–regarding that women’s SA/DP increase during pregnancy compared to the period before and after pregnancy and compared to levels among nulliparous women [1–6, 8]. In contrast, during periods not close to pregnancy, women who gave birth had lower SA/DP than their counterparts who remained nulliparous, suggesting a possible health selection into pregnancy [1, 3–6].

In several occupations within the educational and care branches, overall SA levels are particularly high [16, 17, 40, 41]. High emotional and physical job demands [40], low job control, violence or violence threats, and low skill-level jobs have been suggested as potential explanations [22, 23, 42–44], however, other factors might also exist. All such studies have focused on women (and sometimes men) in general and of all ages in these types of jobs. Here, our focus was on a different group, i.e., nulliparous women of reproductive age who did or did not give birth during the study period. We found that, after taking sociodemographic factors into account, SA/DP levels were similar to those of women in middle socioeconomic status categories, as defined by occupation, from our previous study based on a cohort involving all nulliparous women, aged 16–39 years and residing in Sweden 31 December 2004, and whom we categorized based on four occupational hierarchical classes [4]. Finding that SA/DP values did not stand out in this cohort, as compared to other occupational groups, may relate to the young age of our study population. To our knowledge, only one study by Sydsjö and associates has investigated SA levels in pregnancy in women working in the branches we studied; they reported higher SA levels during pregnancy among women employed in healthcare than in other branches in Norway, but not in Sweden [8].

In Sweden, work organization, psychosocial and physical working conditions have been suggested to be better in the private than in the public sector [29, 30]. Further, attitudes and norms towards SA may traditionally have been more permissive in the public than in the private sector. Two reports by the Swedish Social Insurance Agency involving analyses of the Swedish workforce aged 16–69 years, found higher levels of SA in the public than in the private sector; these differences disappeared when considering differences between sectors in demographic composition of the workforce (and thus partly in health) or differences in organizational size [29, 45]. In contrast, a Norwegian study found no differences in SA between sectors [46]. However, none of the studies included information on DP, even if part-time DP was possible in the studied country. The similarities we found in SA/DP among women in the private and the public sectors may be explained–similarly to the findings of the Swedish Social Insurance Agency [29, 45]–by our inclusion criteria, i.e., a relatively homogeneous population in terms of sex and age, and by standardizing for further demographic factors in the analyses. Moreover, the lack of differences by sector in our study may be related to the relatively high level of regulations in the education and care branches, which, in turn may result in more similar psychosocial and physical working conditions across sectors than in the overall population and consequently more comparable SA/DP levels among young women working in these branches [33].

In this large explorative register-based study we had no information on work characteristics, work organization, detailed organizational characteristics or similar and thus had no possibility to explore the importance of such factors for the sector-specific associations [46]. Traditionally, the public sector has been regarded as more permissive with respect to SA, but after the 1990s any differences may have decreased due to the decentralization and the partial privatization of education and care, rationalization, and an increased profit-orientation in the private sector, with potential implications for differences in SA levels between the public and the private sectors [46, 47].

The main study strengths involve including not only a sample, but all women fulfilling the inclusion criteria, the high-quality register data [35, 48], the use of both the MBR and the NPR registers to identify women who gave birth, the administrative data that precludes the possibility of recall bias and allow following all study participants, and that several covariates that previous studies have found important for SA and DP and childbirth were included in the analyses. There might, of course, be several other factors, at different structural levels, than those we were able to include, and which may be relevant to consider in future studies. For instance, this includes risk factors, confounders or effect modifiers of the studied association, e.g. family support, unpaid and paid working conditions (physical, psychosocial, work-hours, shift-work, time for commuting to work etc.), type of work contract, size of workplace, personal resilience, access to and quality of healthcare, welfare systems, childcare system, economic situation, female employment frequency, as well as categories of providers of care (e.g. hospitals, primary healthcare, care of elderly or of persons with disabilities at home or in specialized care centers etc.) and educational services (preschool or school, extracurricular activities etc.). Hopefully, our study will inspire others to conduct such studies. The lack of information on SA spells <15 days can be seen as both a limitation and a strength. The strength relates to only having information on SA due to more severe conditions, but a limitation as some SA days were missed. However, the shorter SA spells represent a smaller number of all SA days. Another limitation involves not knowing whether some women changed sectors or occupations during follow-up. A further limitation is that some of the women in the cohort might have given birth before immigrating to Sweden. We tried to minimize this misclassification in three ways. All who according to MBR and NPR had given birth before 2005 were excluded. All who gave birth in 2005 and thereafter, who according the MBR had given birth before, were excluded. As the MBR covers up to 99% of births, we had such information for most women who gave birth. The third way was to only include women who had lived in Sweden during at least three years before inclusion, that is in 2002–2004. As countries vary largely in their social insurance systems, quality and coverage of health care, organization and working conditions of employees in the educational and care branches, it is unclear to what extent our findings generalize to other contexts.

In conclusion, we found that nulliparous women working in educational or care branches and giving birth to one child or more children during the study period had, except for the year before childbirth, comparable or lower mean combined SA and DP days than women who remained nulliparous. The women who had more than one birth had the lowest mean number of SA/DP days. Importantly, we observed no substantial differences in these patterns among women working in the public or private sectors.
